# Quantification of the Tissue Sodium Concentration in the Human Calf at 7T With Reduced Acquisition Time: Influence of the Nominal Spatial Resolution

**DOI:** 10.1002/nbm.70365

**Published:** 2026-07-26

**Authors:** Jordan M. Höhn, Tobias Wilferth, Lena V. Gast, Teresa Gerhalter, Christoph Kopp, Michael Uder, Armin M. Nagel

**Affiliations:** ^1^ Institute of Radiology, University Hospital Erlangen Friedrich‐Alexander‐Universität ErlangenNürnberg (FAU) Erlangen Germany; ^2^ Siemens Healthcare AG Forchheim Germany; ^3^ Department of Neurology Medical University of Graz Graz Austria; ^4^ Department of Nephrology and Hypertension Friedrich‐Alexander‐Universität Erlangen‐Nürnberg (FAU) Erlangen Germany; ^5^ Division of Medical Physics in Radiology German Cancer Research Center (DKFZ) Heidelberg Germany

**Keywords:** 7 Tesla, quantitative MRI, skeletal muscle, sodium (^23^Na) MRI, spatial resolution

## Abstract

To evaluate the repeatability and consistency of rapid quantitative ^23^Na MRI of the human calf, an acquisition‐weighted stack‐of‐stars (AW‐SOSt) sequence was implemented on a 7T MRI system and used for all measurements. Three variants, differing only in nominal in‐plane spatial resolution (2.5, 5.0, and 7.5 mm) and corresponding acquisition times (8:04, 4:02, and 2:41 min), while maintaining a constant slice thickness of 15 mm, were employed for imaging the calf skeletal muscle. Quantitative consistency across resolutions and repeatability was evaluated in simulations and consecutive measurements of 10 healthy volunteers. The apparent tissue sodium concentration (aTSC) was determined using a postprocessing pipeline consisting of a *B*_0_, *B*_1_, relaxation, and partial volume correction (PVC). Deviations of the determined aTSC from simulated ground truth were below 6.0%. The simulated coefficient of variation (CV) improved with decreasing spatial resolution from 1.1% to 0.9%. The in vivo findings matched the simulation results. The CV improved with increasing voxel size, decreasing from 2.7% at 2.5‐mm resolution to 2.0% at 7.5‐mm resolution. Measurements remained highly consistent across different in‐plane resolutions, with a small increase in aTSC (≈0.2 mM) observed as a difference between lower resolutions (7.5 mm) and the highest resolution (2.5 mm). All applied protocols showed good repeatability and high consistency between the protocols. aTSC quantification using low‐resolution ^23^Na MRI showed improved repeatability, whereas in vivo measurements showed no effect on quantitative accuracy. This study demonstrates that low‐resolution ^23^Na MRI with short acquisition times combined with PVC could be a practical alternative to commonly used “high‐resolution” techniques with long acquisition times for quantifying aTSC in calf muscle tissue.

## Graphical abstract

Fast ^23^Na MRI of the human calf at 7T was achieved by reducing nominal spatial resolution. Shorter measurement times can improve clinical feasibility of ^23^Na MRI while preserving reliable quantification of apparent tissue sodium concentration through partial volume, relaxation, and *B*_0_/*B*_1_ corrections. Stable aTSC estimates across resolutions and improved repeatability at lower resolutions support the potential use of fast, low‐resolution ^23^Na MRI for quantitative assessment of skeletal muscle in clinical research settings.

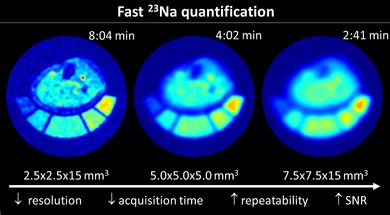

## 1 Introduction

Sodium ions (Na^+^) play a crucial role in maintaining fluid balance, nerve impulse transmission, and muscle function in the body [1]. Sodium (^23^Na) MRI has been established as a noninvasive method to investigate the apparent tissue sodium concentration (aTSC) in human calf muscle, and various studies reported significant alterations in the ion distribution for specific pathologies like diabetes or myopathies. The magnitude of these alterations depends on the specific pathology and disease severity, but reported differences are typically on the order of several millimolar (≈5–15 mM), corresponding to relative increases of approximately 25%–75% compared to healthy control groups [2, 3, 4, 5].

^23^Na MRI is limited by the inherently low MR sensitivity of the ^23^Na nucleus and its low in vivo concentrations [6]. The resulting low signal‐to‐noise ratio (SNR)—which, in skeletal muscle, is approximately 11,000–30,000 times lower than in ^1^H MRI [7, 8]—substantially restricts clinical research applications by necessitating long acquisition times (typically approximately 8 min).

Due to the short ^23^Na relaxation times, center‐out radial readout schemes are advantageous for ^23^Na MRI, as they enable ultrashort echo times and immediate sampling of the k‐space center. Independently, the anisotropic structure of skeletal muscle favors anisotropic sampling strategies that match the geometry of the imaged volume. Therefore, anisotropic radial trajectories such as acquisition‐weighted stack‐of‐stars (AW‐SOSt) are well suited for ^23^Na skeletal muscle imaging. In the AW‐SOSt sequence, center‐out, density‐adapted radial sampling is performed in‐plane, whereas the slice direction is encoded using Cartesian phase encoding (Figure S1). Compared with fully isotropic 3D radial approaches [9] (e.g., 3D‐DA‐RAD), this hybrid encoding improves acquisition efficiency while maintaining the ultrashort echo times required for ^23^Na imaging. Alternative approaches include Cartesian gradient echo (GRE)‐based sequences, which have been applied to calf muscle aTSC quantification at anisotropic resolutions of 3 × 3 × 30 mm^3^ and 3.9 × 3.9 × 20 mm^3^ [5, 10, 11]; however, their longer echo times (TE ~ 2 ms) render them less suitable for the fast‐relaxing ^23^Na signal [11].

Together with improved hardware capabilities, such as increased magnetic field strength, center‐out ultrashort echo time sequences enabled quantitative ^23^Na MRI with high repeatability and reproducibility at 7T, using spatial resolutions of 2.5 × 2.5 × 15.0 mm^3^ [12, 13, 14]. The measurement times of these protocols are ≥ 8 min, which poses a practical barrier to clinical translation and limits the feasibility of repeated or combined measurements within a single session. Because many ^23^Na MRI studies of the human calf muscle focus on quantifying the aTSC over larger regions‐of‐interests (ROIs) [6], high spatial resolution may not be required. However, a reduction of the nominal spatial resolution implicates stronger partial volume effects (PVE), which might introduce a quantitative bias and decrease the repeatability of the aTSC estimation.

The objective of this work was to investigate consistency of aTSC quantification across different in‐plane resolutions and repeatability across repeated measurements using an AW‐SOSt sequence with in‐plane resolutions ranging from 2.5–7.5 mm, while reducing acquisition times from 8:04 to 2:41 min. In particular, the influence on aTSC of advanced postprocessing including *B*_0_ and *B*_1_ correction as well as partial volume correction (PVC), and relaxation correction was evaluated for different spatial resolutions after each correction step.

## 2 Methods

All MR measurements were conducted on a whole‐body 7T MR system (MAGNETOM Terra. X, Siemens Healthcare, Erlangen, Germany) using a dual‐tuned ^23^Na/^39^K leg radiofrequency (RF) coil (RAPID Biomedical, Rimpar, Germany) for ^23^Na MRI and a dual‐tuned ^1^H/^31^P leg RF coil (RAPID Biomedical, Rimpar, Germany) for acquiring high‐resolution ^1^H MR data sets for muscle tissue segmentation. All measurements performed on 10 healthy volunteers (five males and five females, 36 ± 13 years) were approved by the local Ethical Committee of the Faculty of Medicine in Erlangen, on March 16, 2021, 21‐61_1‐B, and all volunteers provided informed written consent prior to the scan.

### 2.1 Measurement Protocol

First, anatomical ^1^H T_1_‐weighted data sets for segmentation of the muscles, the subcutaneous fat and blood vessels were acquired using a Cartesian 3D‐FLASH sequence (TR = 8.1 ms, FA = 6°, TE = 3.57 ms, *t*_*meas*_ = 1:34 min and a nominal spatial resolution of 1 × 1 × 5 mm^3^) and the ^1^H/^31^P RF coil. Afterwards, the RF coil was changed to a ^23^Na/^39^K RF coil without repositioning of the patient by leaving the examined calf on a mount suitable for both RF coils. *B*_0_ shimming was performed based on a ^23^Na B_0_ map (TR = 50 ms/TE = 0.3/5.0 ms/FA = 80°/*t*_*meas*_ = 1:00 min) [15] using the constrained regularized pseudo‐inversion algorithm [16].

Subsequently, three ^23^Na data sets were acquired using the AW‐SOSt readout scheme (Figure S1), which is well suited for imaging of fast relaxing nuclei with intrinsically low SNR. The center‐out readout provides short echo times, whereas density‐adapted in‐plane sampling and an anisotropic Cartesian readout in *z* direction maximize SNR efficiency [17]. Nominal spatial in‐plane resolution of 2.5/5.0/7.5 mm was chosen, satisfying the Shannon‐Nyquist criterion for half spokes, with the corresponding number of spokes per star (252/126/84) yielding *t*_*meas*_ = 8:04/4:02/2:41 min (TR = 120 ms, TE = 0.3/11.3 ms, *t*_*RO*_ = 10 ms, *t*_*Dwell*_ = 26 μs, FA = 90°, and nominal spatial resolution in *z* direction 15 mm). The number of samples per spoke (384), as well as the number of partitions in *z* direction (16), was kept constant (Table S1). Both echoes are acquired within the same TR following a single excitation pulse. After the first readout, a rewinder gradient returns k‐space sampling to the isocenter, enabling a second density‐adapted readout at a later echo time, followed by another rewinder and a spoiler gradient in slice direction. To assess the repeatability of the aTSC determination, the three ^23^Na measurements were repeated immediately after the initial acquisitions without repositioning the subject.

### 2.2 ^23^Na Data Processing and Concentration Estimation

**FIGURE 1:**
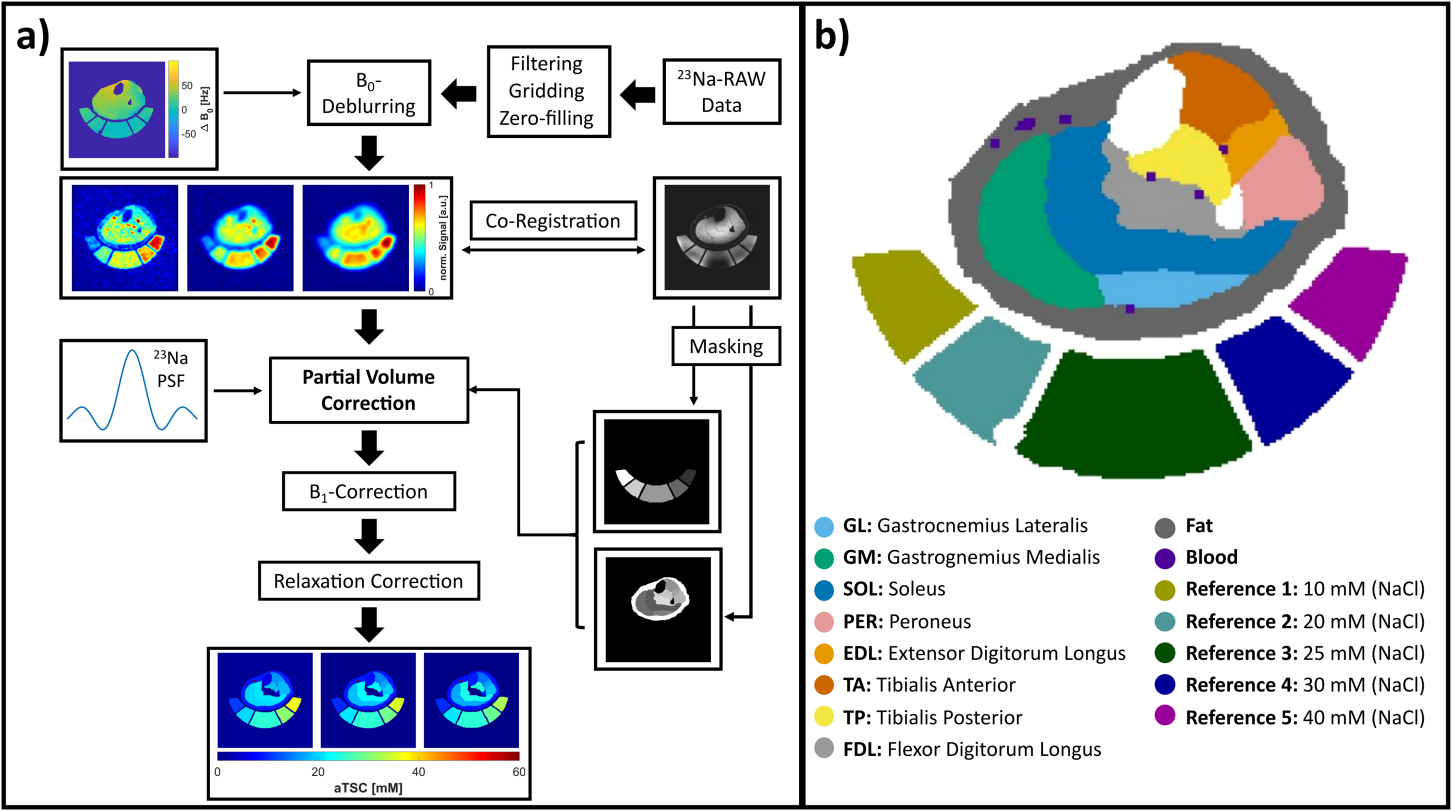
(a) Postprocessing workflow of ^23^Na MRI data. After acquiring an ^1^H image and ^23^Na images with three different resolutions. The ^23^Na images are reconstructed and for *B*_0_ corrected; then, the proton image is registered to the sodium images. Masks are created for muscles, subcutaneous fat, blood vessels, and references using the DAFNE semiautomatic segmentation tool. Sodium images undergo partial volume correction using these masks. Values are then adjusted with premeasured *B*_1_ correction factors (average FA per ROI) and average ^23^Na relaxation values for muscle at 7T, yielding average aTSC values per ROI. In (b), an overview of the segmented ROIs is given for the tissue, as well as the reference phantoms with the respective concentration of NaCl in mM.

The postprocessing workflow is schematically shown in Figure 1.

All ^23^Na images were reconstructed using a nonuniform fast Fourier transformation (NUFFT) [18]. In addition, a correction for nonlinearities of the gradients in the outer FOV, a *B*_0_ correction [19], and a Hamming filter to reduce Gibbs' ringing artifacts and to increase SNR [20, 21, 22] were applied to the k‐space. The weighting for each k‐space value was calculated by the equation $H\left(\left|k\right|\right)=0.54+0.46\cdot \cos\left(2\pi \cdot \frac{|k|}{\text{kmax}\;}\right)$ [22]. The *B*_0_ map was calculated from the phase difference of the dual‐echo acquisition (*ΔTE* = 11 ms). Based on the resulting off‐resonance range, the k‐space data were corrected for a set of discrete frequency offsets, reconstructed separately, and subsequently combined in image space by selecting, on a pixel‐wise basis, the reconstruction corresponding to the closest local *B*_0_ value. This process is shown in detail in Figure S2. The k‐space data were zero‐filled to a resolution of 1 × 1 × 5 mm^3^ (resulting in a matrix size of 200 × 200 × 48 with zero‐filling‐factor of 2.5/5.0/7.5 in‐plane and three in slice direction) to match the nominal spatial resolution of the ^1^H image data sets obtained to draw masks for the PVC.

To correct the ^23^Na signal intensity for PVE, masks were drawn on the registered ^1^H image. Segmentation was performed for the soleus (SOL), gastrocnemius lateralis (GL), gastrocnemius medialis (GM), tibialis anterior (TA), tibialis posterior (TP), peroneus (PER), extensor digitorum longus (EDL), flexor digitorum longus (FDL), subcutaneous fat, blood vessels, and reference phantoms in the central 16 slices of the 200 × 200 × 48 image matrix, centered at the level of maximum transverse calf muscle cross‐sectional area, using the semiautomatic segmentation tool Dafne [23]. To limit manual effort, segmentation was restricted to this central slab (80 mm), which covers the region of interest for quantitative analysis.

The tissue and reference masks were then registered on each ^23^Na image data set for all examined in‐plane resolutions. The ^23^Na MRI image data and the binary masks were used to perform a PVC [24].

The region spread function (RSF) was calculated by folding each segmented mask with the point spread function (PSF). Due to the limited resolution in slice direction (16 partitions), Gibbs ringing occurs at the slab boundaries, particularly for the sharp edges of the binary masks. To minimize its influence, only eight of the 16 slices were used for the GTM calculation. The output of this PVC approach is a corrected mean signal value for the central eight slices of every binary mask. A full overview of the workflow of the PVC is shown in Figure S3.

To reduce the measurement time, the chosen TR results in a slight *T*_1_ weighting. Combined with the rapid biexponential ^23^Na relaxation of transverse magnetization in muscle tissue—contrasting with the slower monoexponential ^23^Na relaxation in the pure saline solution of the reference phantoms—this requires correction for both *T*_1_ and *T*_2_* relaxation to minimize relaxation‐induced bias in aTSC values:

(1) $$c_{\mathrm{ref}}=\left(1-\exp\left(-\frac{\mathrm{TR}}{T_{1}}\right)\right)\exp\left(-\frac{\mathrm{TE}}{T_{2}^{*}}\right)$$

(2) $$c_{\text{tissue}}=\left(1-\exp\left(-\frac{\mathrm{TR}}{T_{1}}\right)\right)\cdot \left(0.6\exp\left(-\frac{\mathrm{TE}}{T_{2,s}^{*}}\right)+0.4\exp\left(-\frac{\mathrm{TE}}{T_{2,l}^{*}}\right)\right)$$

For the correction, literature values of the muscle‐specific relaxation times (T*_2,s_/T*_2,l_ = 3.0/28.0 ms and *T*_1_ = 30.0 ms [25]) and of the pure saline solution of the reference phantoms (*T*_2_* = 56 ms and *T*_1_ = 56.7 ms [26]) were used. These values have also been applied in previous ^23^Na MRI calf muscle studies [3, 11, 13]. These values are also summarized in Table S2.

To account for transmit/receive field (*B*_1_^+/−^) inhomogeneities within the reference phantoms, constant correction factors were applied for each compartment to improve the accuracy of the aTSC quantification. Because *B*_1_^+^ and *B*_1_^−^ are not identical in our setup, factors for the combined effects of transmitter and receiver inhomogeneities were derived from phantom measurements by Gast et al. [13], because the same hardware setup was used. All the tubes in the reference phantom, as well as an additional bottle, were filled with the same saline solution and subsequently imaged. ROIs were drawn for the tubes of the reference phantom and the bottle; then, a PVC was used to calculate corrected mean values of each ROI. As the ^23^Na concentration was the same for all reference tubes and the bottle, the average signal of each compartment is expected to be identical; therefore, any observed deviations were attributed to *B*_1_^+/−^ field inhomogeneities. To correct for these deviations, the inverse signal intensities of each ROI were used as correction factors compensating.

^23^Na quantification was performed using a five‐compartment external reference phantom filled with saline solutions of known NaCl/KCl concentrations (10/240, 20/210, 25/180, 30/150, and 40/120 mM) shown in Figure S4. The phantom was positioned beneath the subject's lower leg within a dedicated coil holder, ensuring a fixed relative geometry and mechanically constrained placement via holder geometry and rubber padding.

A linear regression model of the form S(c(^23^Na)) = a · c(^23^Na) + b was used to describe the relationship between mean reference ROI signal intensity and known sodium concentration. The resulting calibration curve was inverted and applied to the corrected in vivo signal intensities to obtain aTSC values.

In summary, aTSC quantification was performed through a sequential correction and calibration pipeline. Reconstructed ^23^Na images were first corrected for *B*_0_ inhomogeneities, gradient nonlinearities, and partial volume effects. Signal intensities were then extracted from tissue and reference regions of interest and corrected for *T*_1_/*T*_2_* relaxation effects as well as *B*_1_^+^/*B*_1_^−^ inhomogeneities of the reference phantom.

### 2.3 ^23^Na SNR Estimation

The relative theoretical SNR between different resolutions was estimated using Equation (3). Because the readout bandwidth (*t*_*Dwell*_ = 26 μs; *t*_*RO*_ = 10 ms) and the number of samples per spoke were kept constant across all three investigated protocols, changes in the total measurement time (*t*_*meas*_) arose solely from variations in the number of acquired spokes, which were selected according to the Nyquist criterion.

(3) $$\frac{\mathrm{SN}R_{\mathrm{res}}}{\mathrm{SN}R_{2.5\;\mathrm{mm}}}\propto \frac{\Delta \;x\;\Delta \;y\;\Delta \;z\;\sqrt{N_{\text{Spokes}}\left(\mathrm{res}\right)}}{2.5\;\mathrm{mm}\cdot 2.5\;\mathrm{mm}\cdot 15.0\;\mathrm{mm}\cdot \sqrt{N_{\text{Spokes}}\left(2.5\;\mathrm{mm}\right)}}$$

SNR was additionally estimated from the in vivo data. To avoid spatial bias in the noise distribution introduced by the gradient nonlinearity correction, images were reconstructed without gradient and *B*_0_ correction. The images were segmented into noise‐only regions (outside the calf and reference phantoms) and muscle tissue based on the corresponding masks, which were eroded by approximately 1.5 times the FWHM of the point spread function of the 7.5‐mm protocol to minimize partial volume effects.

Noise statistics were estimated from the noise‐only regions by fitting a Rician probability density function to the intensity histograms, yielding an estimate of the underlying noise parameter *σ* [27]. SNR was subsequently calculated for each subject as the ratio of the mean muscle signal to the estimated noise standard deviation.

### 2.4 Simulations

To validate the postprocessing pipeline, simulations based on in vivo–derived segmentation masks were performed [13, 28, 29]. Each mask was Fourier‐transformed and regridded to the AW‐SOSt trajectory, the *T*_2_* decay was applied to all samples along each spoke, and complex Gaussian noise was added to match the SNR of in vivo measurements. The simulated data were then processed using the same algorithms as the in vivo data [28]. The calf model assigned aTSC values of 20 mM for muscles, 7 mM for fat, and 80 mM for blood, along with the tissue‐specific relaxation parameters used in the PVC. Ten independent noise realizations were generated and processed. Sensitivity to estimate inaccuracies was evaluated by varying tissue specific relaxation parameters (muscle *T*_2,s_*: 1.0–5.0 ms and *ω*_*Q*_ = 50–100 Hz, blood *T*_2_*: 12/18 ms) in the simulation. The resulting datasets were used to generate distributions of simulated aTSC values across noise realizations, spatial resolutions, and parameter variations.

Additionally, the influence of the noise floor in the low‐SNR regime was systematically evaluated using numerical simulations. A digital phantom consisting of four cuboid regions of interest (ROIs; 50 × 50 × 100 mm^3^ each) with ^23^Na concentrations of 10, 20, 30, and 40 mM was generated. Simulations were performed for all three spatial resolutions while varying the noise level over a wide range. For each condition, the mean signal within each ROI was quantified.

To assess noise‐induced (Rician) bias, complex Gaussian noise was added to the raw data (k‐space) level, with its amplitude defined relative to the central k‐space value of the noise‐free simulation to ensure consistent scaling across conditions. Image reconstruction was performed using gridding, followed by the SNR calculation for each respective ROI. For each parameter combination, 10 independent noise realizations were generated to enable averaging and statistical evaluation of the results.

### 2.5 Statistical Analysis

For the statistical analysis of the in vivo data, as well as for the simulation, the coefficient of variation (CV) was calculated to test for repeatability:

(4) $$CV_{\text{aTSC}}=\frac{\mathrm{SD}\left(\text{aTSC}\right)}{\text{mean}\left(\text{aTSC}\right)}$$

For the in vivo evaluation, repeatability was assessed using a test–retest design in which each of the three spatial resolutions was acquired twice consecutively without subject repositioning. The two repeated measurements were performed approximately 15 min apart, and CV values were calculated per subject from the paired acquisitions with identical resolution. A repeated measures ANOVA test was performed on the aTSC values at a 5% level of significance to check for significant changes in aTSC over the tested resolutions.

Normality was assessed using the Shapiro–Wilk test. Sphericity was evaluated using Mauchly's test, and if violated, the Greenhouse–Geisser correction was applied. The coefficient of variation was used as a descriptive, unitless measure of within‐subject repeatability, whereas repeated measures ANOVA was used to assess systematic differences in aTSC across spatial resolutions.

A Bland–Altman analysis was performed to assess agreement between repeated measurements. For each spatial resolution, mean aTSC values across all muscles were calculated per subject for both repeated acquisitions. The Bland–Altman plot was constructed by plotting the difference between repeated measurements (Measurement 2 − Measurement 1) against their corresponding mean values. Limits of agreement were defined as the mean difference ±1.96 standard deviations.

## 3 Results

### 3.1 Simulation‐Based Validation of Postprocessing

**FIGURE 2:**
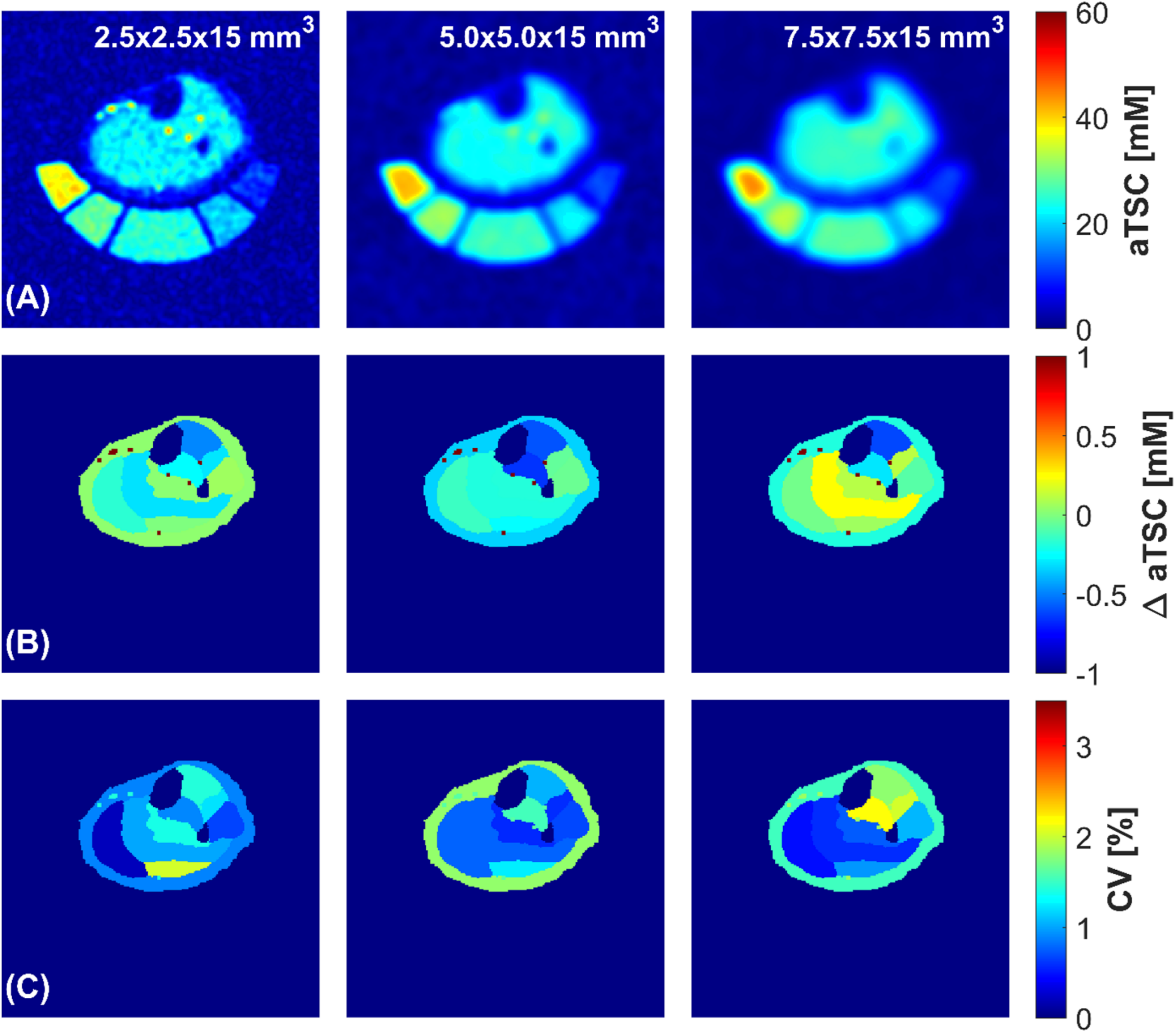
Simulated ^23^Na signal for 2.5‐/5.0‐/7.5‐mm in‐plane resolution. In (A), the intensity values of the three images were already normalized by the references and corrected for relaxation effects, showing concentrations maps in mM. In (B), the mean difference between simulation result and GT (muscles: 20 mM; fat: 7 mM; blood: 80 mM) for each compartment after the PVC. (C) The CV over all noise sets of the simulation in each compartment.

The simulations conducted to verify the accuracy of the postprocessing showed large differences to the ground truth (GT) for estimated aTSC in muscle ROIs before correction of PVE. The mean aTSC was 22.3/23.0/23.7 mM with CV of 0.8/0.6/0.5% over all simulated subjects and noise sets. After correction of PVE, mean aTSC estimates were reduced to 19.8 ± 0.3/19.7 ± 0.3/19.9 ± 0.3 mM with CV of 1.1/0.9/0.9% for the respective resolution 2.5/5.0/7.5 mm averaged over the muscle ROIs of all 10 simulated subjects and their respective 10 noise sets (Figure S5). Figure 2 shows an example of a simulated calf, alongside the results of the corrections applied to each muscle and the deviation from GT.

The variations in the simulated tissue parameters, without adjustment in the correction, resulted in a resolution‐dependent variation in aTSC after postprocessing for both *ω*_*Q*_ and *T*_2,s_* of the muscle tissue (Figure S6).

### 3.2 ^23^Na In Vivo MRI Measurements

aTSC values were determined in 10 healthy volunteers (subject characteristics are summarized in Table S3) for the three examined spatial in‐plane resolutions of 2.5/5.0/7.5 mm. Due to scheduling conflicts, both 5.0‐mm resolution measurements were omitted for Subject 07 and the second 5.0‐mm resolution measurement for Subject 09.

#### 3.2.1 Repeatability

Repeatability was assessed using test–retest measurements acquired approximately 15 min apart without repositioning. CV values decreased with increasing voxel size: 2.7% at 2.5 mm, 2.5% at 5.0 mm, and 2.0% at 7.5 mm resolution. More detailed analysis of the CV for the individual muscles can be found in Table S4. A general drift in aTSC towards lower values was observed in repeated scans (Figure S7).

#### 3.2.2 aTSC Quantification

**FIGURE 3:**
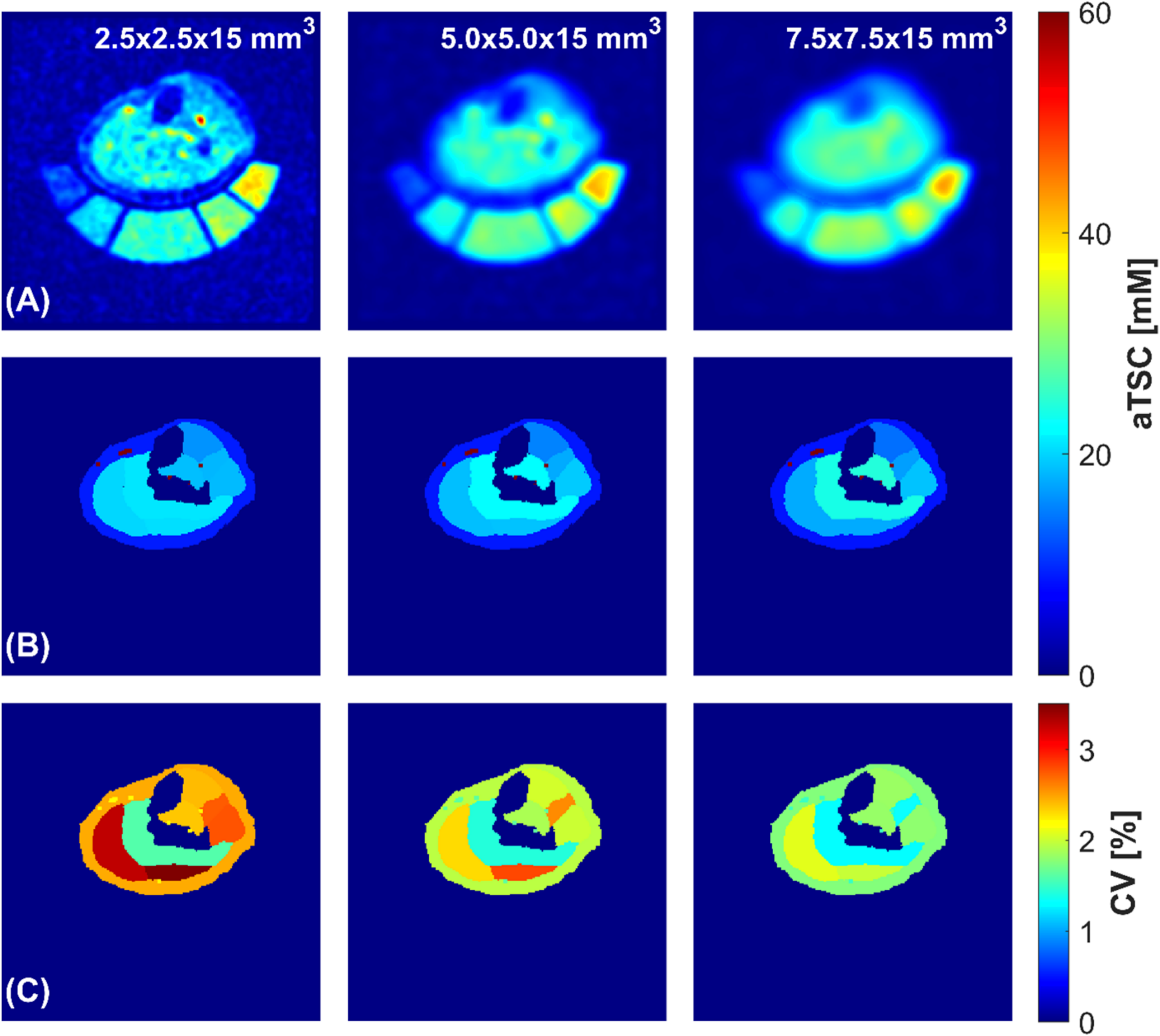
(A) In vivo ^23^Na images normalized to the reference concentrations for the three in‐plane resolutions tested (2.5/5.0/7.5 mm). (B) Exemplary results of the mean aTSC of one volunteer for the respective ROI excluding FDL after the postprocessing corrections filled in the segmentation masks as a constant. (C) CVs of the repetition measurement for each compartment for this subject.

Table 1 summarizes the subject‐wise mean muscle aTSC for each spatial resolution and both measurements. Notably, intrasubject variability between muscle groups was substantial in some cases (e.g., Subjects 04, 07, and 10), with SD of 5.3–7.6 mM. Figure 3 illustrates the results exemplified by subject 01, showing the uncorrected aTSC map, corresponding CVs, and corrected values after postprocessing. Whereas PER, EDL, and TA exhibited small variation between resolutions (SD = 0.7 mM), SOL and TP showed larger discrepancies (SD = 2.0 mM) for this subject. These results are comparable to the other nine subjects (Table 1).

**TABLE 1:** Table of the mean aTSC value over all muscle groups (except FDL) for both measurements for each individual subject after the complete postprocessing scheme.

Subject	Sex	Age (years)	aTSC_2.5_ (mM)	aTSC_5.0_ (mM)	aTSC_7.5_ (mM)
01	W	27	19.5/19.4	19.3/19.6	20.2/20.0
02	M	26	15.4/14.9	15.8/15.6	15.3/15.3
03	M	27	17.9/17.3	18.6/18.0	18.2/17.4
04	M	60	24.4/23.9	25.6/24.5	25.7/25.4
05	W	58	19.0/17.8	18.6/17.6	18.9/18.3
06	M	32	19.3/18.7	19.4/18.7	19.5/19.1
07	W	28	20.5/20.2	−/−	20.9/20.5
08	W	35	21.3/19.9	20.4/19.3	21.0/20.0
09	M	31	25.3/24.2	24.0/−	24.3/23.6
10	W	34	24.1/22.4	25.4/22.9	23.8/22.1

Final mean aTSC values averaged over all muscles (excluding FDL) are summarized in Table 1, ranging from 15.4 to 26.1 mM. In the absence of a ground truth, protocols with lower spatial resolutions were compared to the highest resolution (2.5 mm), which had already been used for several studies prior to this [12, 13, 14]. The voxel‐size‐dependent difference between 7.5‐ and 2.5‐mm resolutions was 0.2 ± 0.6 mM, comparable to the 0.1 ± 0.7 mM difference between 5.0 and 2.5 mm.

#### 3.2.3 Effect of PVC on Quantification

**FIGURE 4:**
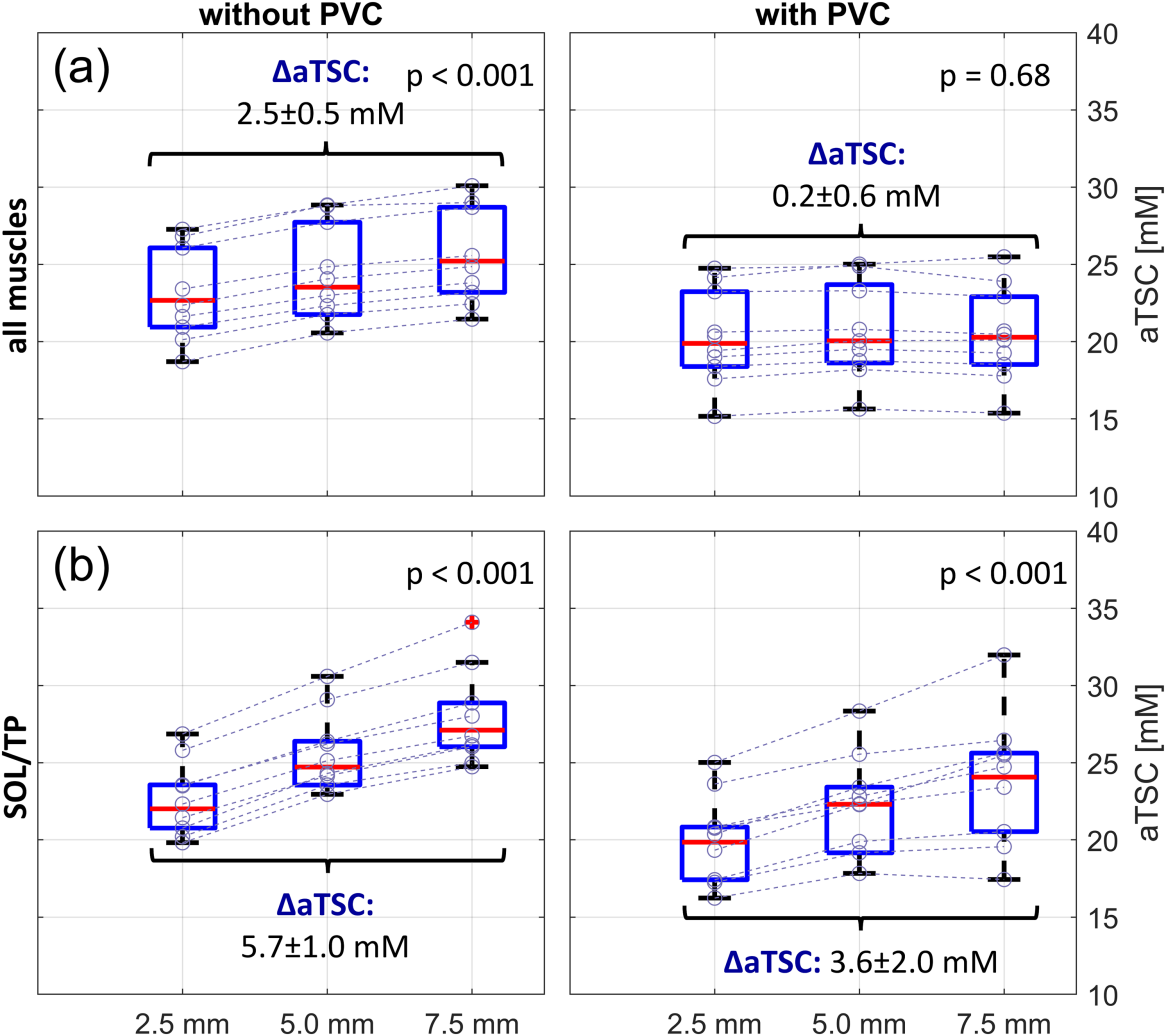
Comparison of the measured mean aTSC values depending on the image resolution of all subjects analysed without corrections (left) and with corrections (right). In (a), the mean values were calculated from all segmented muscles, whereas in (b), only soleus and tibialis posterior were used to determine an average aTSC in the muscle tissue.

Figure 4a presents boxplots of mean aTSC per subject before and after PVC. Without correction, aTSC increased significantly with voxel size (mean difference 2.5 ± 0.5 mM between 2.5 and 7.5 mm, *p* < 0.001).

After PVC, this effect was reduced to 0.2 ± 0.6 mM showing no significant change in aTSC (*p* = 0.67), indicating improved consistency across resolutions. In specific muscle groups such as SOL and TP (Figure 4b), the bias remained pronounced even after correction, with a mean difference of 3.6 ± 2.0 mM (*p* < 0.001), in contrast to other muscles (GL, GM, PER, EDL, and TA), which showed smaller deviations, as well as lower aTSC values for the highest resolution with an average difference (−1.1 ± 0.8 mM, *p* < 0.001). A more detailed overview of the influence of corrections on the quantification can be found in Figure S8.

#### 3.2.4 SNR Evaluation

In vivo SNR estimates for the three spatial resolutions are summarized in Figure S9. A clear increase in SNR with increasing voxel size was observed, with median values of 11.0 ± 1.4 for 2.5‐mm, 33.3 ± 5.1 for 5.0‐mm, and 52.2 ± 7.1 for 7.5‐mm in‐plane resolution. The variability of SNR estimates was higher for the highest spatial resolution, which is likely attributable to residual partial volume effects, particularly from vascular signal contributions that cannot be fully excluded by the automated segmentation masks.

The observed SNR ratios between resolutions deviated from the theoretical estimates, with ratios of approximately 4.6 (7.5/2.5 mm) and 3.0 (5.0/2.5 mm). Overall, the presented analysis should therefore be interpreted as a practical estimate of the SNR regime rather than an exact quantitative measure.

To further assess the influence of SNR on aTSC estimation, additional simulations were performed across a range of noise levels (Figure S10). The results indicate that, within the SNR range observed in vivo, aTSC estimates remain largely unaffected by noise across resolutions. Noticeable deviations were only observed for the highest spatial resolution at very low SNR levels (SNR < 2), where increased variability and bias became apparent.

## 4 Discussion

The primary objective of this study was to evaluate the repeatability and consistency of rapid quantitative ^23^Na MRI of the skeletal muscle using an AW‐SOSt acquisition scheme and whether measurement time can be reduced by exploiting the increased SNR at lower spatial resolutions. Our results demonstrate that aTSC values averaged over large muscle groups remain quantitatively consistent across a threefold change in spatial resolution, despite a substantial reduction in acquisition time (8:04–2:41 min). Although systematic, resolution‐dependent biases were observed at the level of individual muscles, the overall deviation across all muscles was small, indicating that robust quantification is feasible for region‐based analyses. These findings suggest that high spatial resolution is not strictly required for reproducible aTSC measurements when consistent acquisition protocols are used, although the level of anatomical detail influences the extent of resolution‐dependent bias.

The small average deviation in aTSC across spatial resolutions (0.2 ± 0.6 mM) with respect to the highest resolution (2.5 mm) indicates that the reduction in spatial resolution does not introduce a relevant quantitative bias for region‐based analysis. This deviation is well within the expected measurement precision for ^23^Na MRI in skeletal muscle, which is typically on the order of ≥ 1 mM [6]. These findings support the feasibility of reducing acquisition time by lowering spatial resolution without compromising the reliability of aTSC quantification at the level of large muscle groups.

The average aTSC for all three resolutions over all skeletal muscles excluding FDL was generally within the range of the values reported in literature (~15–28 mM) [6, 30, 31, 32, 33, 34]. The acquisition and correction strategies employed in existing aTSC studies differ substantially, complicating direct comparisons between reported values. Cartesian GRE‐based protocols have been applied with strongly anisotropic voxels (e.g., 3 × 3 × 30 mm^3^ [5, 10, 11], 3.9 × 3.9 × 20 mm^3^ [5]), which offer SNR efficiency but at the cost of through‐plane PVEs in anatomically heterogeneous regions. Center‐out radial sequences, including 3D density‐adapted radial (3 × 3 × 15 mm^3^ [3, 11]; 2.5 × 2.5 × 2.5 mm^3^ [4]) and stack‐of‐stars readouts (6 × 6 × 25 mm^3^ [2]; 2.5 × 2.5 × 15 mm^3^ [12]), are particularly suited to ^23^Na imaging due to their short echo times, which reduce *T*_2_* signal loss inherent to fast‐relaxing nuclei. The present work employed an AW‐SOSt at 7 T, combining the SNR benefit of a center‐out trajectory with the efficiency of anisotropic Cartesian encoding in *z* direction [17], at a field strength that provides a substantial SNR advantage over 3 T systems used in several prior studies [3, 10, 11].

With respect to correction schemes, three of the reviewed studies applied no corrections [2, 5, 10], one applied *B*_0_ correction only [3], and one applied relaxation correction and PVC [12], whereas only two studies applied a full correction pipeline comparable to the present work [4, 13]. *B*_0_ and *B*_1_ corrections reduce system‐dependent biases introduced by field inhomogeneities and transmit/receive field variations, which are strongly dependent on coil geometry, field strength, and shimming quality; although the aTSC estimation over an ROI including all muscle groups yielded robust results, the large variations observed in aTSC between muscle groups in some subjects suggest the need for a muscle‐wise aTSC evaluation. Here, FDL was excluded from the analysis due to the fact that this compartment is characterized by a high proportion of fascia, in addition to a significant presence of larger blood vessels surrounding and traversing it. This resulted in substantial variations in the measured aTSC.

The observed repeatability in our measurements (2.7%, 2.5%, and 2.0% for 2.5, 5.0, and 7.5 mm, respectively) is either comparable to or higher than previously reported values in the brain for ^23^Na MRI with 2%–8% scan‐rescan variability at 3T and 7T [35, 36], as well as 15% intrasite variability over 22 weeks in skeletal muscle [37] and differences of 1.7–4.5 mM in aTSC for measurements of skeletal muscle repeated on the same day [4, 10, 13]. The high repeatability of our study is likely attributable to the high field strength (7T) and the short interval (~15 min) between repeated scans. Despite the overall good repeatability, a general drift towards a lower aTSC during the course of the measurement was observed. However, this effect was relatively small (0.8, 0.7, and 0.6 mM for 2.5, 5.0, 7.5 mm, respectively) and might be explained by a physiological decrease in muscle aTSC over time with prolonged supine position [38]. Overall, the low CV values confirm that reducing spatial resolution does not compromise measurement stability and further supports the feasibility of shortening acquisition time without degrading quantitative performance.

The observed consistency of aTSC across spatial resolutions can be explained by the interplay of the applied correction steps and the underlying signal characteristics. *B*_0_ and gradient nonlinearity corrections primarily ensure accurate spatial fidelity of the reconstructed images and are essential for maintaining correct alignment between the ^23^Na data and the anatomical segmentation masks. This alignment is critical for PVC, as registration errors would directly affect the effectiveness of the PVC, and could be improved by double‐tuned ^23^Na/^1^H RF coils. In contrast, *B*_1_^+^/*B*_1_^−^ and relaxation corrections primarily improve the stability and accuracy of the concentration calibration. The *B*_1_ correction compensates for systematic sensitivity differences across the reference compartments. The relaxation correction is particularly important due to the stronger *T*_1_ weighting of the reference phantoms compared to muscle tissue, which would otherwise introduce a substantial bias in the concentration estimation. In addition, the correction for *T*_2_* relaxation increases the measured aTSC. As these corrections are applied uniformly across all spatial resolutions, they do not introduce resolution‐dependent bias. The dominant resolution‐dependent effect therefore arises from PVE, which are mitigated by the PVC approach. These described effects are shown in Figure S8. As spatial resolution decreases, increased signal mixing between tissues occurs. However, the applied PVC compensates for this effect at the level of larger regions, thereby preserving quantitative consistency. Together, these factors explain why robust aTSC quantification can be achieved at lower spatial resolutions despite increased voxel size. This interpretation is further supported by the in vivo SNR analysis, which demonstrated a clear increase in SNR with increasing voxel size. In particular, residual PVE and the scaling of noise contributions may contribute to the observed deviations. Although efforts were made to minimize vascular signal contamination by eroding the tissue masks, small residual effects cannot be fully excluded. Overall, these findings confirm that the increased SNR at lower spatial resolutions compensates for the reduced acquisition time, thereby maintaining stable aTSC quantification despite shorter measurements.

Although region‐based quantification of large ROIs (full muscle tissue) remained robust across resolutions, the analysis of individual muscles revealed the limitations of low‐resolution quantification. The high intensity signal originating from blood vessels (≈81 mM [25]) strongly infiltrated the surrounding voxels at low resolutions influencing the concentration estimation in the muscle. This effect was more pronounced in muscles surrounded by large blood vessels (SOL and TP), and the quantification showed larger deviations between the resolutions, with an average difference between 2.5/7.5 mm in‐plane resolution of ≈4 mM, even after PVC. The residual deviation likely reflects inherent limitations of the PVC, as accurate correction depends on a precise segmentation of the blood vessels, which is itself constrained by the limited resolution of the anatomical ^1^H reference. At this resolution, the ^1^H image is already subject to partial volume effects at the vessel boundaries, and this segmentation uncertainty propagates into the PVC of the ^23^Na image, limiting its ability to fully recover spilled vascular signal. Qualitatively, this observation was confirmed in simulations. However, deviations were smaller, suggesting that inaccuracies in segmentation masks might increase this effect.

In the simulations, estimated aTSC was higher for the 7.5 mm in contrast to the 2.5 mm resolution, but the effect was much weaker than in the in vivo data. This might be caused by slight segmentation errors or deviations between real and assumed tissue parameters in in vivo data for the performed correction. Changing the relaxation parameters (especially *T*_2,s_* = 1–5 ms) in the simulation, while leaving these parameters unchanged in the corrections, generally resulted in deviations of the measured aTSC from the GT due to the deviation in the simulated and corrected relaxation factors resulting from Equations (1) and (2). In addition to this effect, the aTSC measured in GM, GL, TA, EDL, and PER muscles showed dependency on the resolution (7.5 vs. 2.5 mm), whereas the SOL, FDL, and TP muscles did not. This likely reflects changes in the PSF: in muscles surrounded mostly by other muscle tissue (Figure 1b), the effects of the PSF on aTSC balance each other out, whereas muscles adjacent to tissues such as fat/vessels are more affected. This can pose challenges in pathological muscle, such as in dystrophic muscle tissue, because the microscopic tissue composition is typically altered by adipose infiltration, which may compromise models predicated on healthy muscle structure. In this context, ^1^H fat‐fraction maps might be incorporated into the PVE [39, 40]. Additionally, pathological changes such as extracellular or intracellular oedema may alter the relaxation behavior of skeletal muscle tissue as well. However, the results of the simulations show that in the PVC the isolated effects of deviations from GT in the relaxation parameters have only a weak influence on the effective aTSC but that a combination of several effects (nonideal segmentation and deviations in the real and corrected relaxation values of the tissue) could explain the observations of the in vivo measurements.

Even though the quantification of ^23^Na in muscle tissue at very low resolutions already yielded good results, several aspects could still be improved. To improve general reliability, more detailed masks could be created that also include smaller blood vessels. This would further reduce PVE in the surrounding muscle tissue. In addition, measurement time might further be reduced, for example, by under sampling the radial 2D trajectories of the AW‐SOSt, which is possible due to the high SNR at low resolutions, as well as by increasing the measured slice thickness, which was also kept constant in this experiment. However, this approach presents its own challenges, as an investigation into the impact of under sampling artifacts on the quantification would be necessary.

## 5 Conclusion

We successfully demonstrated good repeatability and high consistency between the examined protocols with different spatial in‐plane resolutions. In particular, this work shows that low‐resolution ^23^Na MRI protocols with short acquisition times combined with PVC may be a practical alternative for use in clinical research compared to the commonly used “high‐resolution” techniques with long acquisition times.

## Author Contributions

**Jordan M. Höhn:** methodology, software, validation, formal analysis, investigation, resources, data curation, writing – original draft, visualization, project administration. **Tobias Wilferth:** methodology, resources, writing – review and editing, supervision, project administration. **Lena V. Gast:** conceptualization, resources. **Teresa Gerhalter:** methodology, resources. **Christoph Kopp:** resources. **Michael Uder:** resources. **Armin M. Nagel:** conceptualization, methodology, writing – review and editing, supervision, project administration, funding acquisition.

## Funding

This work was supported by Deutsche Forschungsgemeinschaft (500888779/RU5534).

## Conflicts of Interest

Lena Gast is an employee of Siemens Healthineers International AG. Other than this, the author has no conflicts of interest. The rest of the authors declare no conflicts of interest.

## Supporting information


**Figure S1:** (a) Pulse sequence diagram illustrating the acquisition of acquisition weighted stacks‐of‐stars (AW‐SOSt), combining Cartesian phase encoding in the slice direction with radial center‐out sampling in‐plane. Each “star” consists of a set of radial spokes acquired at a given partition, with angular weighting applied across partitions.
**Figure S2:** Reconstruction workflow with B_0_ deblurring. Starting from the acquired k‐space data, a Hamming filter, density compensation, gridding, gradient correction, and zero‐filling to a reconstructed resolution of 1x1x5 mm^3^ are applied, yielding two‐echo images from which a off‐resonance map is computed (ΔTE = 11 ms). For B_0_ deblurring, the off‐resonance map is partitioned into *n* = 20 equally spaced frequency intervals, and a binary mask is generated for each interval. The mean off‐resonance frequency Δω_off,i_ of each interval is used to demodulate the k‐space data via the corresponding phase term, after which the image is reconstructed and multiplied by its associated mask. The *n* masked sub‐images are finally summed to yield the B_0_‐corrected, deblurred image.
**Figure S3:** The PVC procedure is based on segmented ^1^H‐derived tissue masks registered to the reconstructed ^23^Na data. First, the PSF is computed from the k‐space trajectory, including effects of the applied filter and T2 relaxation weighting. In a second step, RSFs are generated by convolution of the PSF with the corresponding binary tissue masks. Third, the geometric transfer matrix (GTM) is calculated by evaluating the mean overlap between all combinations of masks and RSFs. Finally, the GTM is inverted and applied to the vector of measured mean signal intensities across all regions, yielding partial volume corrected signal estimates for each ROI.
**Figure S4:** Schematic of the reference phantom showing compartment‐specific contents. Each compartment was filled with aqueous solutions of NaCl and K_2_HPO_4_, giving the indicated Na^+^ and K^+^ concentrations (mM).
**Figure S5:** In (a) the resulting aTSC from the simulation with identical simulated and corrected tissue parameters is shown for each muscle group respectively. In (b) the distribution of individual muscle aTSC results over all simulated subjects and noise data sets is shown for the three simulated resolutions as a histogram. The ground truth aTSC of 20 mM is marked in both with a dashed line.
**Figure S6:** In (a), as the quadrupolar interaction frequency (ω_Q_) was varied, the average difference between aTSC values of the 7.5 mm and 2.5 mm resolution after post‐processing was 0.6 mM, with higher values for the 2.5 mm resolution and an increasing SD for higher simulated ω_Q_ values. In contrast, the variation of T_2,l_* of the blood vessels did not introduce a resolution‐dependent bias on the quantitative values (b). In (c), the variation of T_2,s_* of the muscle tissue demonstrated an increase in aTSC for higher resolutions, with values reaching up to 1.8% higher at T_2,s_* = 5 ms. When analysed by muscle group, SOL, TP and FDL muscles consistently demonstrated higher aTSC at 7.5 mm (≈0.35 mM), which was largely unaffected by T_2,s_*. Conversely, the remaining muscles exhibited a clear dependence on T_2,s_*, resulting in a quantitative bias associated with resolution.
**Figure S7:** Bland–Altman plots for the average muscle aTSC of each subject on the x‐axis and the difference of the measurement and its repetition for the resolutions of 2.5 mm, 5.0 and 7.5 mm. The dashed lines depict the ±1.96 SD and the solid lines the mean difference between the measurement and its repetition.
**Figure S8:** aTSC across subjects for the three acquired resolutions (2.5 mm, 5.0 mm, 7.5 mm) under applied reconstruction and quantification corrections. Boxes show the subject‐wise distribution. The five correction stages are cumulative: “No correction” denotes the baseline reconstruction; “Grad. Corr.” adds gradient trajectory correction; “B_0_ Corr.” additionally compensates for off‐resonance‐induced blurring; “relax. + B_1_ Corr.” further accounts for T_1_/T_2_* relaxation losses and transmit‐ and receive‐field inhomogeneities; and “PVC” finally adds the partial volume correction. Within each correction group, the three resolutions are plotted side by side and color‐coded as indicated in the legend, allowing both the effect of each added correction and the residual resolution dependence of aTSC to be assessed.
**Figure S9:** SNR estimates derived from in vivo data across three spatial resolutions (2.5/5.0/7.5 mm). Tissue masks obtained from the corresponding ^1^H images were used to define signal regions (muscle tissue excluding fat and blood vessels) and noise regions. To reduce partial volume effects, all masks were eroded by 0.5xFWHM of the point spread function. Noise characteristics were estimated by fitting the magnitude signal in background regions to a Rician distribution with Rayleigh bias correction. SNR was calculated as the ratio of the mean signal within the muscle ROIs to the estimated noise level. Boxplots summarize the distribution of SNR values across subjects, with individual measurements overlaid as scatter points. Median SNR values were 11.0 ± 1.4 for 2.5 mm, 33.3 ± 5.1 for 5.0 mm, and 52.2 ± 7.1 for 7.5 mm spatial resolution.
**Figure S10:** Simulated datasets comprising four compartments (10, 20, 30, and 40 mM) were generated for all three spatial resolutions. Complex Gaussian noise was added in k‐space at different levels relative to the maximum signal amplitude to achieve varying SNR conditions. For each resolution and noise level, ten independent noise realizations were simulated, reconstructed, and analysed. (a) Representative reconstructed images for low, intermediate, and high SNR conditions across the three spatial resolutions. (b) Mean reconstructed signal within ROIs as a function of SNR on the left and the CV of the mean ROI signal over the 10 noise iterations as a function of SNR. ROIs were eroded by 0.5xFWHM of the point spread function to reduce partial volume effects. SNR was estimated by fitting the noise distribution to a Rician model with Rayleigh bias correction and defining SNR as the ratio of the mean signal within the ROI to the estimated noise level. Red boxes indicate the approximate SNR range observed in the in vivo measurements.
**Table S1:** Sequence parameters of the ^23^Na‐AW‐SOSt protocols.
**Table S2:** Table of the relaxation parameters used for corrections [1, 2].
**Table S3:** Subject demographics including sex, age, weight, and body mass index (BMI). Weight and BMI were obtained from the pre‐examination questionnaire and were not measured directly; therefore, these values should be considered approximate.
**Table S4:** Table of the in vivo muscle specific CV averaged over all subjects.

## Data Availability

The data that support the findings of this study are not publicly available.
