# Supplementary material for: Quantification of the Tissue Sodium Concentration in the Human Calf at 7T With Reduced Acquisition Time: Influence of the Nominal Spatial Resolution
Source: NMR Biomed. 2026 Jul 26;39(9):e70365. doi: 10.1002/nbm.70365 (PMC13402084; doi:10.1002/nbm.70365)
Supplement: Supplementary file 1 — Figure S1: (a) Pulse sequence diagram illustrating the acquisition of acquisition weighted stacks‐of‐stars (AW‐SOSt), combining Cartesian phase encoding in the slice direction with radial center‐out sampling in‐plane. Each “star” consists of a set of radial spokes acquired at a given partition, with angular weighting applied across partitions. Figure S2: Reconstruction workflow with B0 deblurring. Starting from the acquired k‐space data, a Hamming filter, density compensation, gridding, gradient correction, and zero‐filling to a reconstructed resolution of 1x1x5 mm3 are applied, yielding two‐echo images from which a off‐resonance map is computed (ΔTE = 11 ms). For B0 deblurring, the off‐resonance map is partitioned into n = 20 equally spaced frequency intervals, and a binary mask is generated for each interval. The mean off‐resonance frequency Δωoff,i of each interval is used to demodulate the k‐space data via the corresponding phase term, after which the image is reconstructed and multiplied by its associated mask. The n masked sub‐images are finally summed to yield the B0‐corrected, deblurred image. Figure S3: The PVC procedure is based on segmented 1H‐derived tissue masks registered to the reconstructed 23Na data. First, the PSF is computed from the k‐space trajectory, including effects of the applied filter and T2 relaxation weighting. In a second step, RSFs are generated by convolution of the PSF with the corresponding binary tissue masks. Third, the geometric transfer matrix (GTM) is calculated by evaluating the mean overlap between all combinations of masks and RSFs. Finally, the GTM is inverted and applied to the vector of measured mean signal intensities across all regions, yielding partial volume corrected signal estimates for each ROI. Figure S4: Schematic of the reference phantom showing compartment‐specific contents. Each compartment was filled with aqueous solutions of NaCl and K2HPO4, giving the indicated Na+ and K+ concentrations (mM). Figure S5: In [file NBM-39-e70365-s001.docx]

## **Supporting Information**

**Quantification of the tissue sodium concentration in the human calf at 7T with reduced acquisition time: Influence of the nominal spatial resolution**

Jordan M. Höhn^a^, Tobias Wilferth^a^, Lena V. Gast^a,b^, Teresa Gerhalter^a,c^, Christoph Kopp^d^, Michael Uder^a^, Armin M. Nagel^a,e^

^a^ Institute of Radiology, University Hospital Erlangen, Friedrich-Alexander-Universität Erlangen-Nürnberg (FAU), Erlangen, Germany

^b^ Siemens Healthcare GmbH, Erlangen, Germany

^c^ Department of Neurology, Medical University of Graz, Graz, Austria

^d^ Department of Nephrology and Hypertension, Friedrich-Alexander-Universität Erlangen-Nürnberg (FAU), Erlangen, Germany

^e^ Division of Medical Physics in Radiology, German Cancer Research Center (DKFZ), Heidelberg, Germany

**
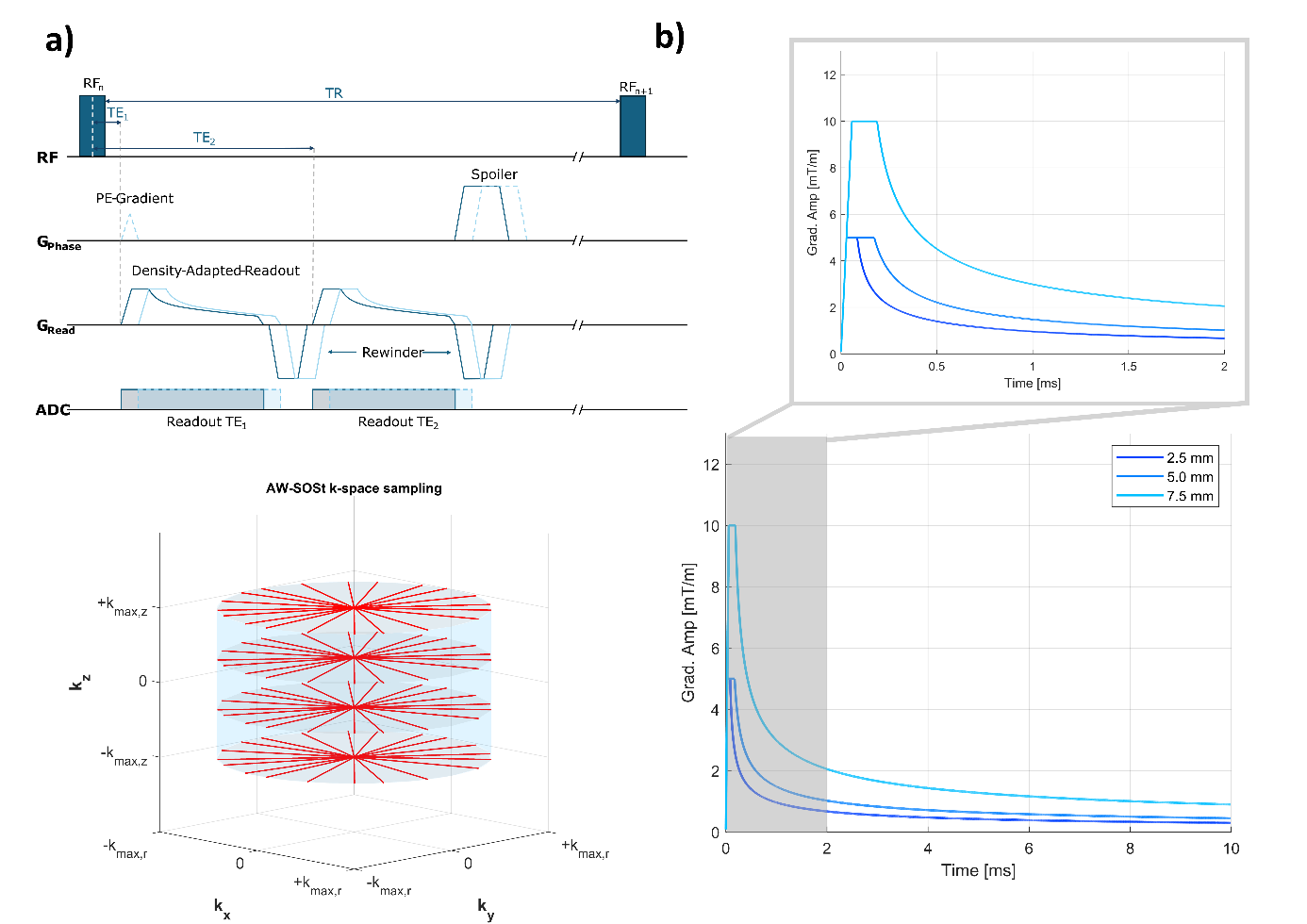
**

**Supplemental Figure S1:** *a) Pulse sequence diagram illustrating the acquisition of acquisition weighted stacks-of-stars (AW-SOSt), combining Cartesian phase encoding in the slice direction with radial center-out sampling in-plane. Each “star” consists of a set of radial spokes acquired at a given partition, with angular weighting applied across partitions.*

*Below the in-plane radial sampling pattern with angularly interleaved spokes across partitions, forming a stack-of-stars trajectory along the k_z_-direction. The acquisition scheme enables anisotropic spatial resolution with efficient k-space coverage and ultrashort echo times.*

*In b) the gradient trajectories are shown for the 3 resolutions, color-coded as named in the legend. Above it the gradient trajectories are shown for the first 2 ms of the readout (gray area) with the same color-coding. The parameters (t_0_, G_0_) for each resolution can be found in Table S1.*

*
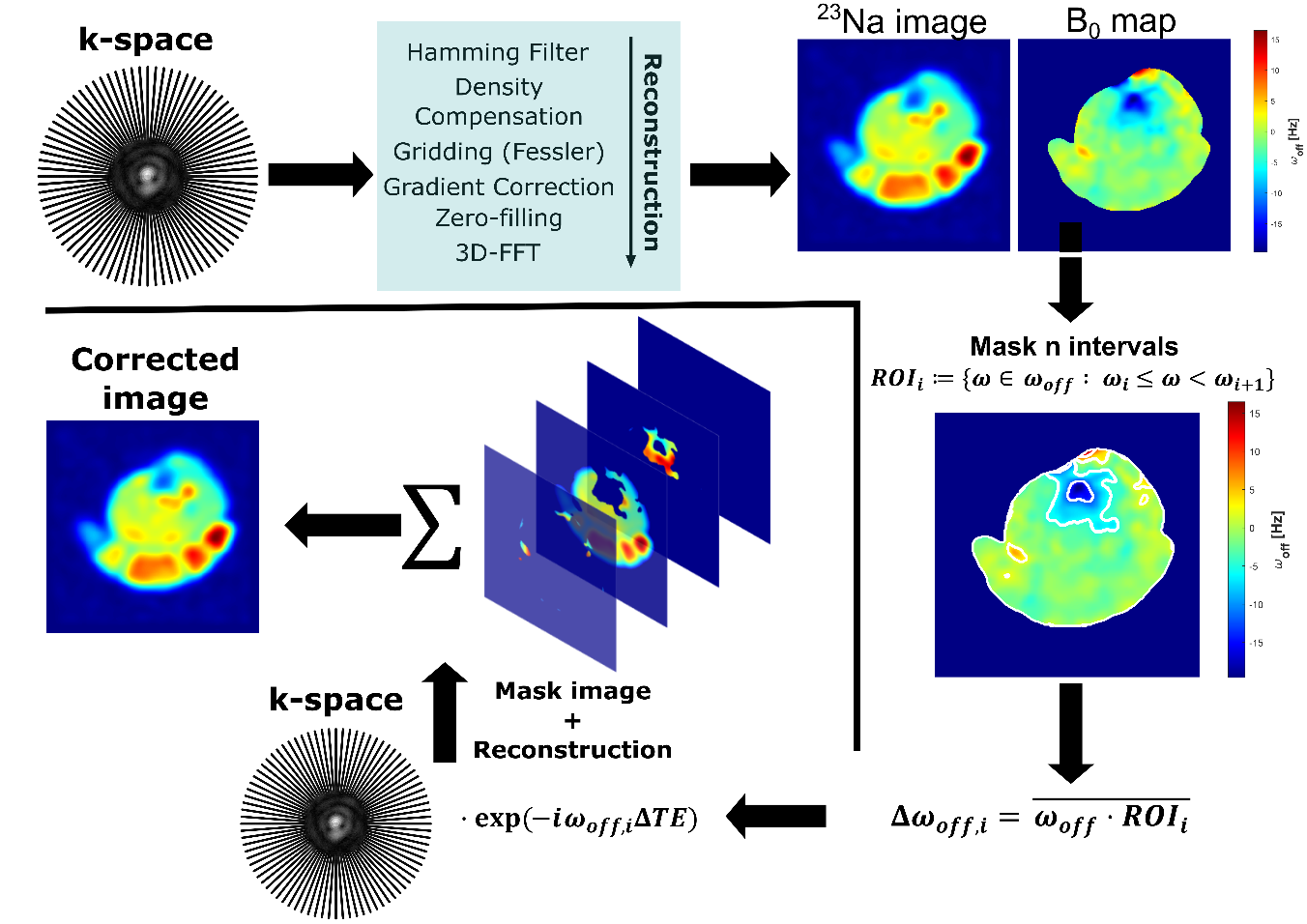
*

**Supplemental Figure S2:**  *Reconstruction workflow with B₀ deblurring. Starting from the acquired k-space data, a Hamming filter, density compensation, gridding, gradient correction, and zero-filling to a reconstructed resolution of 1x1x5 mm³ are applied, yielding two-echo images from which a off-resonance map is computed (ΔTE = 11 ms). For B_0_ deblurring, the off-resonance map is partitioned into n = 20 equally spaced frequency intervals, and a binary mask is generated for each interval. The mean off-resonance frequency Δω_off,i_ of each interval is used to demodulate the k-space data via the corresponding phase term, after which the image is reconstructed and multiplied by its associated mask. The n masked sub-images are finally summed to yield the B₀-corrected, deblurred image.*

**
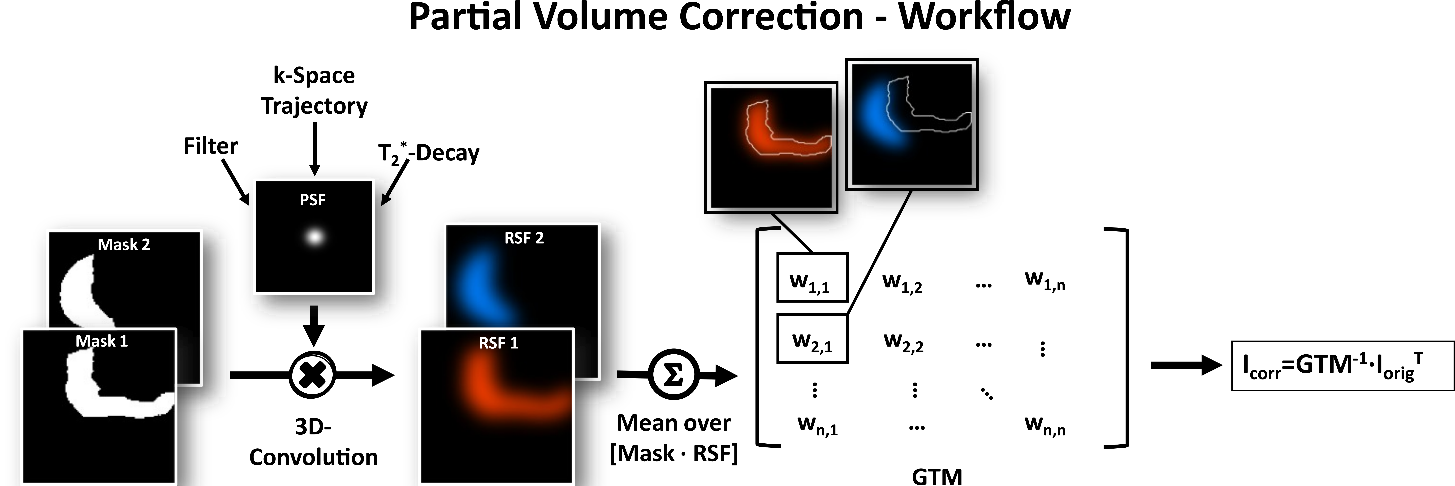
**

**Supplemental Figure S3:**  *The PVC procedure is based on segmented ¹H-derived tissue masks registered to the reconstructed ²³Na data. First, the PSF is computed from the k-space trajectory, including effects of the applied filter and T2 relaxation weighting. In a second step, RSFs are generated by convolution of the PSF with the corresponding binary tissue masks. Third, the geometric transfer matrix (GTM) is calculated by evaluating the mean overlap between all combinations of masks and RSFs. Finally, the GTM is inverted and applied to the vector of measured mean signal intensities across all regions, yielding partial volume corrected signal estimates for each ROI.*

**
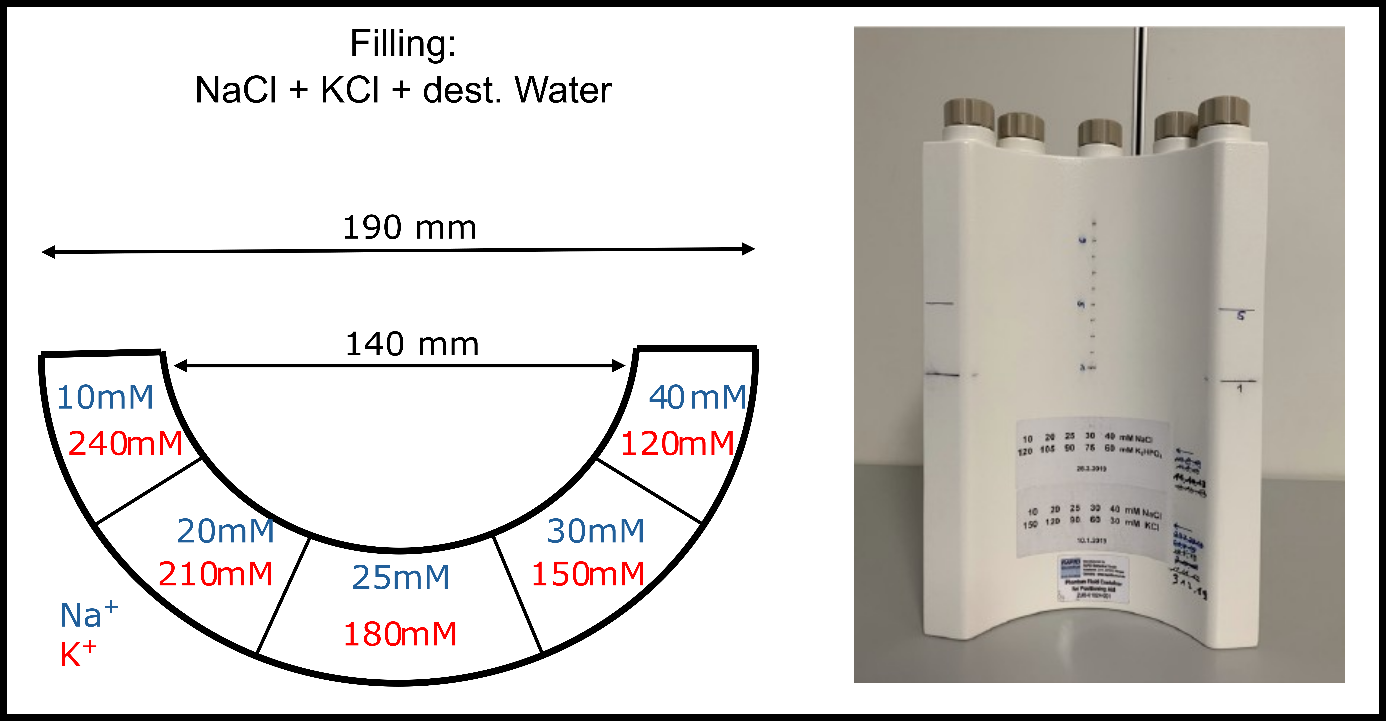
**

**Supplemental Figure S4:** *Schematic of the reference phantom showing compartment-specific contents. Each compartment was filled with aqueous solutions of NaCl and K_2_HPO_4_, giving the indicated Na^+^ and K^+^ concentrations (mM).*

**
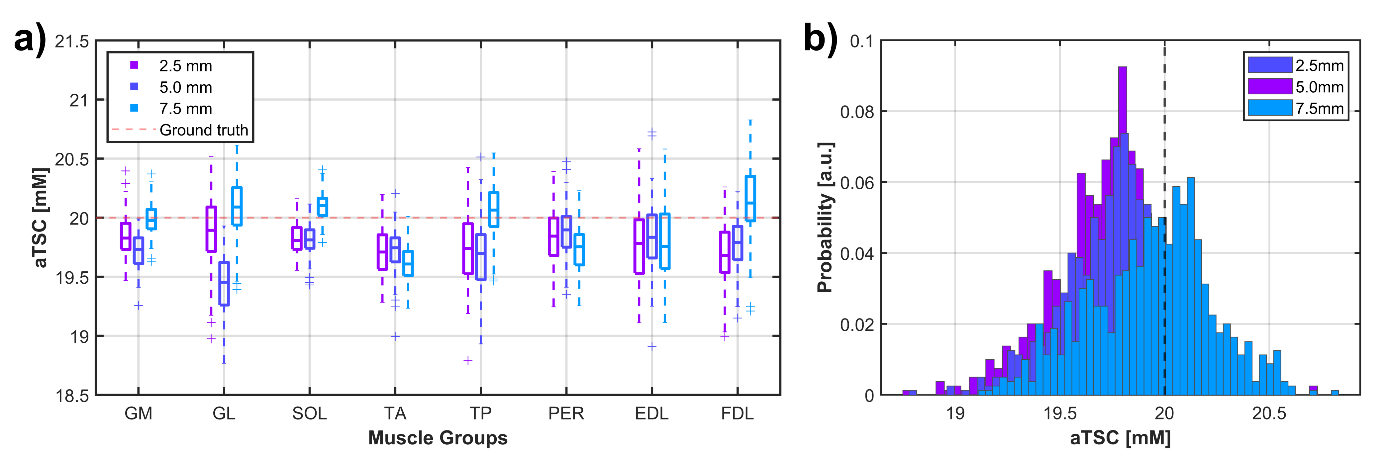
**

**Supplemental Figure S5:** *In a) the resulting aTSC from the simulation with identical simulated and corrected tissue parameters is shown for each muscle group respectively. In b) the distribution of individual muscle aTSC results over all simulated subjects and noise data sets is shown for the three simulated resolutions as a histogram. The ground truth aTSC of 20 mM is marked in both with a dashed line.*

**
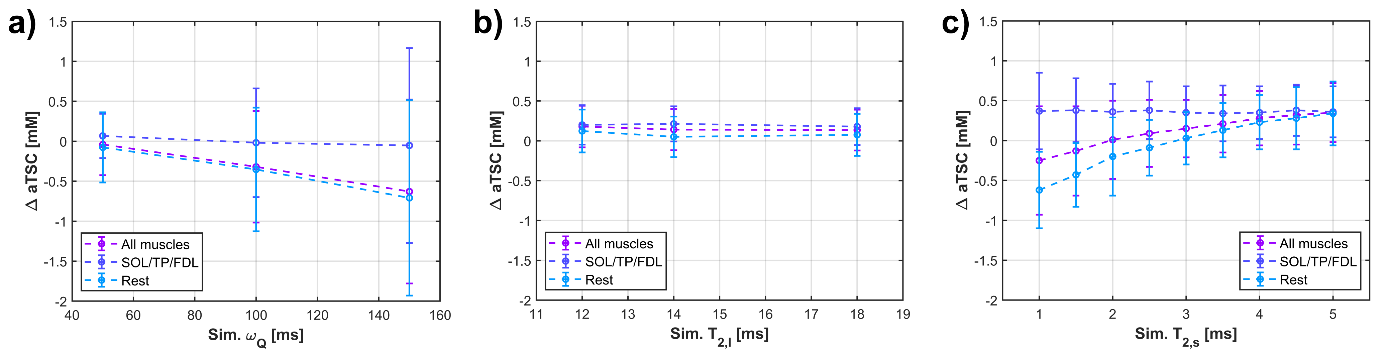
**

**Supplemental Figure S6:** *In a), as the quadrupolar interaction frequency (ω_Q_) was varied, the average difference between aTSC values of the 7.5 mm and 2.5 mm resolution after post-processing was 0.6 mM, with higher values for the 2.5 mm resolution and an increasing SD for higher simulated ω_Q_ values. In contrast, the variation of T_2,l_^*^ of the blood vessels did not introduce a resolution-dependent bias on the quantitative values (b). In c), the variation of T_2,s_^*^ of the muscle tissue demonstrated an increase in aTSC for higher resolutions, with values reaching up to 1.8% higher at T_2,s_^*^ = 5 ms. When analysed by muscle group, SOL, TP and FDL muscles consistently demonstrated higher aTSC at 7.5 mm (≈0.35 mM), which was largely unaffected by T_2,s_^*^. Conversely, the remaining muscles exhibited a clear dependence on T_2,s_^*^, resulting in a quantitative bias associated with resolution.*

**
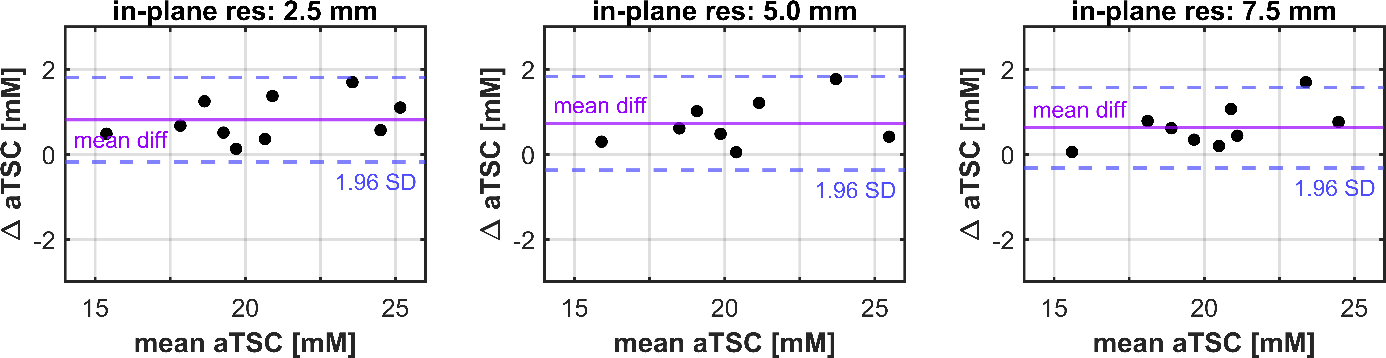
**

**Supplemental Figure S7:** *Bland-Altman plots for the average muscle aTSC of each subject on the x-axis and the difference of the measurement and its repetition for the resolutions of 2.5 mm, 5.0 and 7.5 mm. The dashed lines depict the ±1.96 SD and the solid lines the mean difference between the measurement and its repetition.*


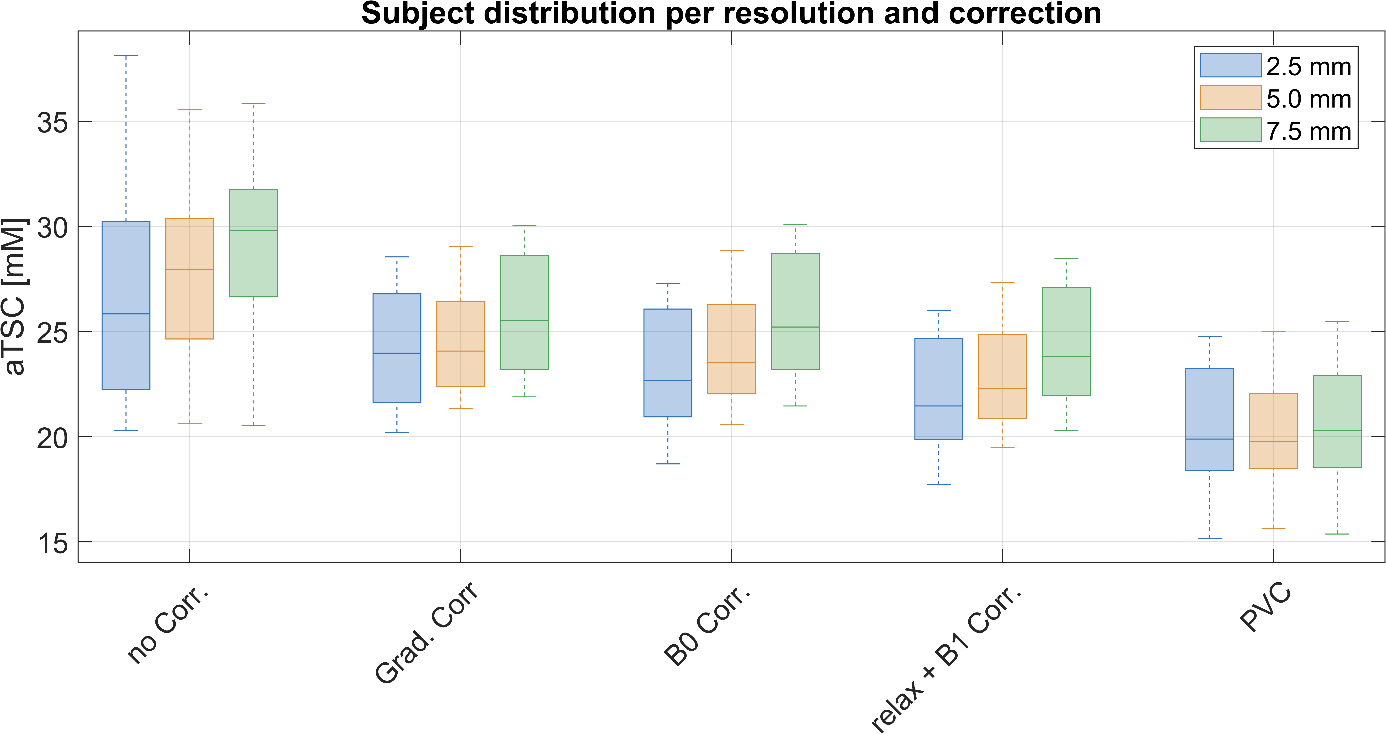


**Supplemental Figure S8:**  *aTSC across subjects for the three acquired resolutions (2.5 mm, 5.0 mm, 7.5 mm) under applied reconstruction and quantification corrections. Boxes show the subject-wise distribution. The five correction stages are cumulative: "No correction" denotes the baseline reconstruction; "Grad. Corr." adds gradient trajectory correction; "B_0_ Corr." additionally compensates for off-resonance-induced blurring; "relax. + B_1_ Corr." further accounts for T_1_/T_2_^*^ relaxation losses and transmit- and receive-field inhomogeneities; and "PVC" finally adds the partial volume correction. Within each correction group, the three resolutions are plotted side by side and color-coded as indicated in the legend, allowing both the effect of each added correction and the residual resolution dependence of aTSC to be assessed.*


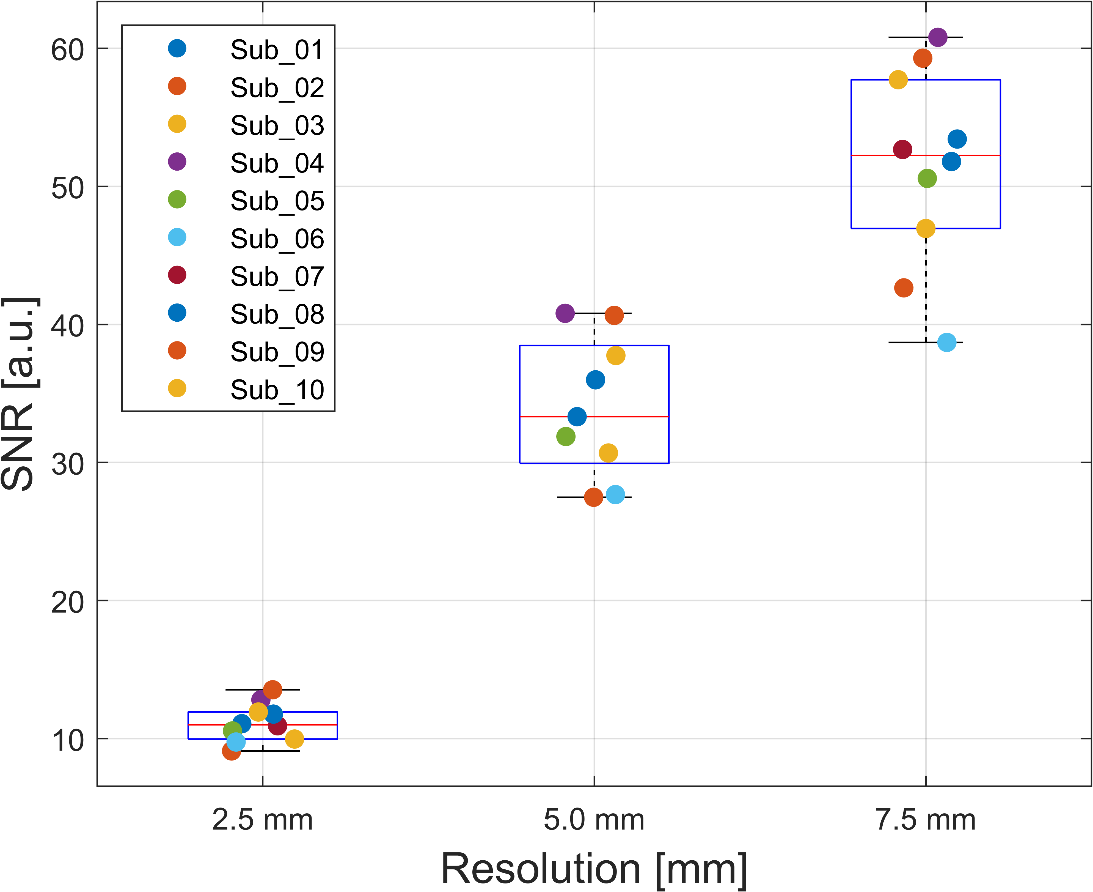


**Supplemental Figure S9:** *SNR estimates derived from in vivo data across three spatial resolutions (2.5/5.0/7.5 mm). Tissue masks obtained from the corresponding ¹H images were used to define signal regions (muscle tissue excluding fat and blood vessels) and noise regions. To reduce partial volume effects, all masks were eroded by 0.5xFWHM of the point spread function.*

*Noise characteristics were estimated by fitting the magnitude signal in background regions to a Rician distribution with Rayleigh bias correction. SNR was calculated as the ratio of the mean signal within the muscle ROIs to the estimated noise level.*

*Boxplots summarize the distribution of SNR values across subjects, with individual measurements overlaid as scatter points. Median SNR values were 11.0 ± 1.4 for 2.5 mm, 33.3 ± 5.1 for 5.0 mm, and 52.2 ± 7.1 for 7.5 mm spatial resolution.*

**
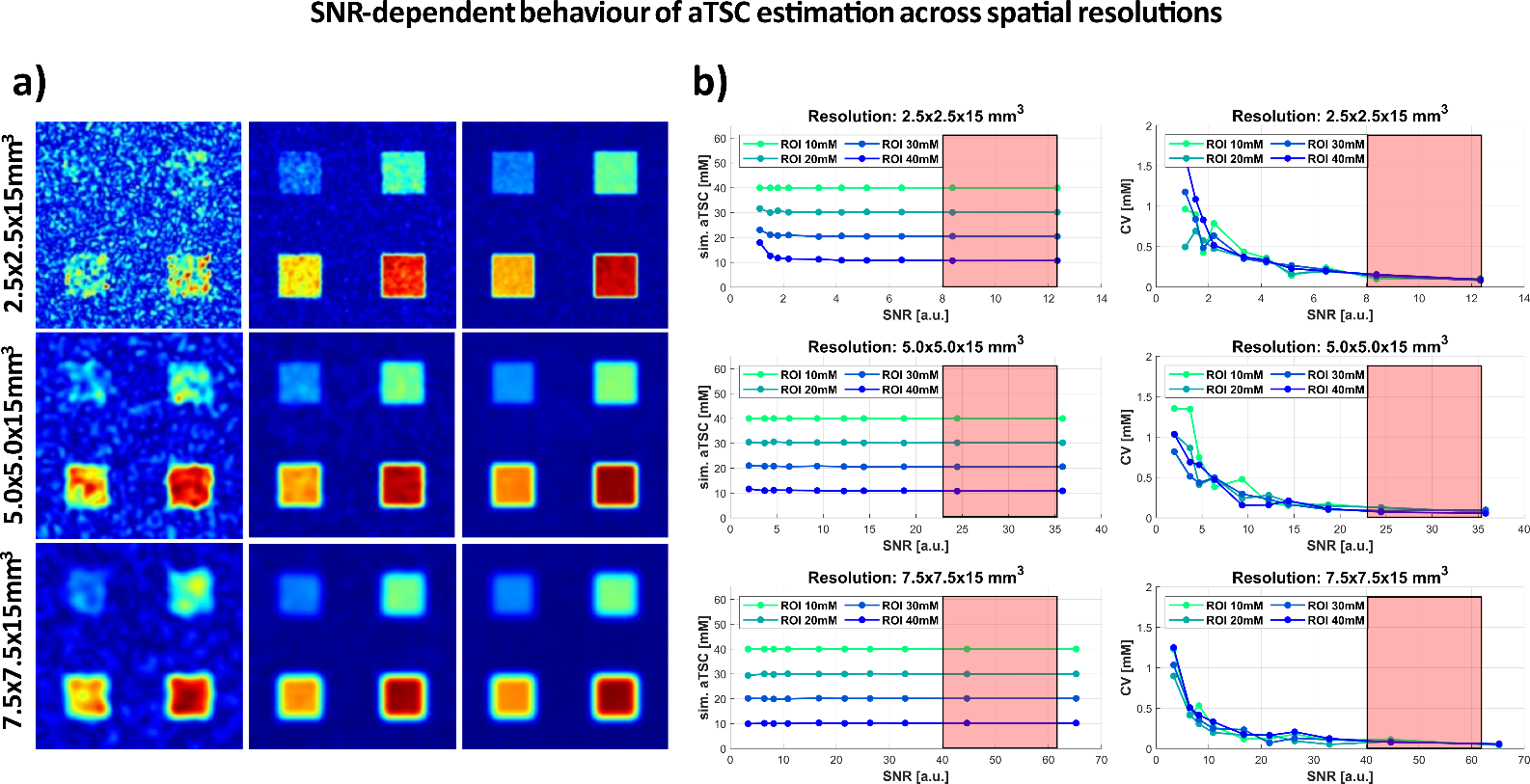
**

**Supplemental Figure S10:** *Simulated datasets comprising four compartments (10, 20, 30, and 40 mM) were generated for all three spatial resolutions. Complex Gaussian noise was added in k-space at different levels relative to the maximum signal amplitude to achieve varying SNR conditions. For each resolution and noise level, ten independent noise realizations were simulated, reconstructed, and analysed.*

*(a) Representative reconstructed images for low, intermediate, and high SNR conditions across the three spatial resolutions.*

*(b) Mean reconstructed signal within ROIs as a function of SNR on the left and the CV of the mean ROI signal over the 10 noise iterations as a function of SNR. ROIs were eroded by 0.5xFWHM of the point spread function to reduce partial volume effects.*

*SNR was estimated by fitting the noise distribution to a Rician model with Rayleigh bias correction and defining SNR as the ratio of the mean signal within the ROI to the estimated noise level. Red boxes indicate the approximate SNR range observed in the in vivo measurements.*

**Supplemental Table S1:** *Sequence parameters of the ^23^Na-AW-SOSt protocols.*

|  | Protocol 1 | Protocol 2 | Protocol 3 |
| --- | --- | --- | --- |
| Resolution | 2.5x2.5x15.0 mm^3^ | 5.0x5.0x15.0 mm^3^ | 7.5x7.5x15.0 mm^3^ |
| TE | 0.3/11.3 ms | 0.3/11.3 ms | 0.3/11.3 ms |
| TR | 120 ms | 120 ms | 120 ms |
| FA | 90° | 90° | 90° |
| t_read_ | 10 ms | 10 ms | 10 ms |
| t_0_ | 0.085 ms | 0.174 ms | 0.188 ms |
| G_0_ | 10 mT/m | 5 mT/m | 5 mT/m |
| N_Samples_ | 384 | 384 | 384 |
| N_Spokes_ | 252 | 126 | 84 |
| N_Partitions_ | 16 | 16 | 16 |
| t_meas_ | 8:04 min | 4:02 min | 2:41 min |

**Supplemental Table S2:** *Table of the relaxation parameters used for corrections [1, 2].*

|  | T_1_ | T^*^_2,s_ | T^*^_2,l_ |
| --- | --- | --- | --- |
| Muscle | 30.0 ms | 3.0 ms | 28.0 ms |
| Fat | 30.0 ms | 3.0 ms | 28.0 ms |
| Blood | 49.5 ms | - | 14.7 ms |
| Saline Solution | 56.7 ms | - | 56.0 ms |

1. Lott, J., et al., *Corrections of myocardial tissue sodium concentration measurements in human cardiac (23) Na MRI at 7 Tesla.* Magn Reson Med, 2019. **82**(1): p. 159–173.

2. Wilferth, T., et al., *(23)Na MRI of human skeletal muscle using long inversion recovery pulses.* Magn Reson Imaging, 2019. **63**: p. 280–290.

**Supplemental Table S3:** *Subject demographics including sex, age, weight, and body mass index (BMI). Weight and BMI were obtained from the pre-examination questionnaire and were not measured directly; therefore, these values should be considered approximate.*

| Subject | Sex | Age [a] | Weight [kg] | BMI [kg/m^2^] |
| --- | --- | --- | --- | --- |
| 01 | w | 27 | 69 | 23.6 |
| 02 | m | 26 | 81 | 22.0 |
| 03 | m | 27 | 80 | 24.2 |
| 04 | m | 60 | 80 | 26.1 |
| 05 | w | 58 | 55 | 17.8 |
| 06 | m | 32 | 73 | 23.6 |
| 07 | w | 28 | 85 | 35.8 |
| 08 | w | 35 | 50 | 17.9 |
| 09 | m | 31 | 88 | 23.9 |
| 10 | w | 34 | 68 | 22.2 |

**Supplemental Table S4:** *Table of the in vivo muscle specific CV averaged over all subjects.*

| CV [%] | 2.5 mm | 5.0 mm | 7.5 mm |
| --- | --- | --- | --- |
| GM | **3.35** ± 1.02 | **2.95** ± 1.36 | **2.64** ± 1.10 |
| GL | **3.80** ± 1.82 | **3.27** ± 1.49 | **2.38** ± 1.07 |
| SOL | **1.45** ± 1.26 | **1.40** ± 1.13 | **0.99** ± 0.99 |
| TA | **2.51** ± 1.04 | **2.60** ± 0.99 | **1.95** ± 1.27 |
| TP | **2.26** ± 1.86 | **2.24** ± 1.19 | **1.56** ± 1.11 |
| PER | **2.61** ± 1.45 | **2.27** ± 1.83 | **2.08** ± 1.69 |
| EDL | **2.57** ± 1.72 | **2.52** ± 1.69 | **2.05** ± 2.00 |
